# How Competent Are Healthcare Professionals in Working According to a Bio-Psycho-Social Model in Healthcare? The Current Status and Validation of a Scale

**DOI:** 10.1371/journal.pone.0164018

**Published:** 2016-10-18

**Authors:** Dominique Van de Velde, Ank Eijkelkamp, Wim Peersman, Patricia De Vriendt

**Affiliations:** 1 Department of Occupational Therapy, Artevelde University College, Ghent, Belgium; 2 Faculty of Medicine and Health Sciences, Department of Rehabilitation Sciences and Physiotherapy, Occupational Therapy Programme, Ghent University, Ghent, Belgium; 3 Faculty of Medicine and Health Care Sciences, Department of Family Medicine and Primary Health Care, Ghent University, Ghent, Belgium; 4 Department Gerontology and Frailty in Ageing (FRIA) Research Group, Vrije Universiteit Brussel, Brussels, Belgium; IRCCS E. Medea, ITALY

## Abstract

**Background:**

Over the past decades, there has been a paradigm shift from a purely biomedical towards a bio-psycho-social (BPS) conception of disability and illness, which has led to a change in contemporary healthcare. However, there seems to be a gap between the rhetoric and reality of working within a BPS model. It is not clear whether healthcare professionals show the necessary skills and competencies to act according to the BPS model.

**Objective:**

The aim of this study was (1) to develop a scale to monitor the BPS competencies of healthcare professionals, (2) to define its factor-structure, (3) to check internal consistency, (4) test-retest reliability and (5) feasibility.

**Design and Setting:**

Item derivation for the BPS scale was based on qualitative research with seven multidisciplinary focus groups (n = 58) of both patients and professionals. In a cross-sectional study design, 368 healthcare professionals completed the BPS scale through a digital platform. An exploratory factor analysis was performed to determine underlying dimensions. Statistical coherence was expressed in item-total correlations and in Cronbach’s α coefficient. An intra-class-correlation coefficient was used to rate the test-retest reliability.

**Results:**

The qualitative study revealed 45 items. The exploratory factor analysis showed five underlying dimensions labelled as: (1) networking, (2) using the expertise of the client, (3) assessment and reporting, (4) professional knowledge and skills and (5) using the environment. The results show a good to strong homogeneity (item-total ranged from 0.59 to 0.79) and a strong internal consistency (Cronbach’s α ranged from 0.75 to 0.82). ICC ranged between 0.82 and 0.93.

**Conclusion:**

The BPS scale appeared to be a valid and reliable measure to rate the BPS competencies of the healthcare professionals and offers opportunities for an improvement in the healthcare delivery. Further research is necessary to test the construct validity and to detect whether the scale is responsive and able to detect changes over time.

## Introduction

Over the past decades, there has been a paradigm shift from a purely biomedical towards a bio-psycho-social (BPS) conception of disability and illness, which has led to a change in contemporary healthcare [1–4]. A BPS model is defined as a model including both the person and the illness in the reasoning process of the healthcare professional [5]. The origin of this BPS model lies in the awareness that a purely biomedical model does not serve and fulfil the needs in contemporary healthcare, specifically because healthcare nowadays faces an important demographic and epidemiological transition, confronting us with the challenge of the growing group of patients with chronic diseases and the growing group of patients with multimorbidity [6]. Focusing on the cure and the eradication of the disease is not suitable, and other approaches focusing on the highest possible level of health is proposed. Therefore, a BPS approach is needed. Indeed, to provide a basis for understanding the determinants of health, including disability, a model must also take into account ‘the patient, the social context in which he lives, and the complementary system devised by the society to deal with the disruptive effects of the illness or the disability’ [5]. Since this gradual shifting towards a more BPS paradigm, concepts such as client-centred-practice, inclusion, shared decision making, coaching and self-management has gained more importance in healthcare to such an extent that these concepts are taken for granted and serve as guiding principles in practice. Taking these principles for granted, however, contains the insidious risk to step into unforeseen errors and pitfalls when planning and executing the intervention according to the philosophical background of the BPS model.

Notwithstanding the fact that the BPS model was described for the first time in 1977 by Engel [5], it took decades to convince healthcare professionals to employ the BPS model into their clinical reasoning [7–9]. One of the catalysing factors in the adoption was the publication of the International Classification of Functioning, Disability and Health (ICF) [10] by the World Health Organisation (WHO), since the ICF relies on a BPS model integrating two opposing models: the medical and the social model (ICF, p. 20). The ICF attempted to provide a coherent view of different perspectives of health, where health is not merely seen as biological, but also containing psychological and social aspects. Consequently, guided and recommended by WHO, healthcare providers worldwide nowadays strive to enable people to perform daily activities and resume participation in important life roles after being affected by injury or disease [11,12]. These efforts, however, require a broad set of competencies ranging from being an expert in short-term recovery, being an expert in coaching the patient towards an autonomous and independent individual in society [9,13] and require therefore also a focus on human functioning in all its aspects. This latter can be offered by the ICF and the accompanying terminology. Different studies and discourses on the topic show that there seems to be a gap between the rhetoric and the reality of working within a BPS model [9,12,14–16]. Despite the widely used concept of the BPS model and the existence of different theories and classifications, of which the ICF is the most broadly accepted [17–24], the operationalisation of the BPS model remains fragmented. A serious amount of inhibiting factors exist, such as (1) difficulties in engaging the patient in the therapy process [24–26], (2) not knowing how and when to shift from biomedical issues to the psycho-social issues [7], (3) not enough familiarity with the underlying theoretical paradigm and how it evolved since its origin [14,26] and (4) problems in how to prioritise one aspect (biological, psychological and social) over the other [27–29].

In order to enhance practitioners’ effectiveness in reasoning and acting according to a BPS model in daily practice the first step should be analysing the current state of the professionals’ BPS practice. To do so, an appropriate measurement instrument is essential. Unfortunately, no comprehensive measurement instrument comprising all relevant BPS items existed. Therefore the research group set out to develop a measurement instrument to rate the level of BPS practice of healthcare professionals It was the aim to develop a self-report instrument.

Furthermore, it was the objective of this study (1) to report on the development of the instrument; and to establish (2) the factor structure, (3) internal consistency, (4) test-retest reliability and (5) feasibility.

## Methods

### Step 1: development of the scale and item derivation

Item derivation for the scale was based on qualitative research within a grounded theory tradition [30]. A focus group methodology [31] with 7 focus groups with both clients (n = 12) (consumers of healthcare with varying disabilities and healthy individuals), healthcare professionals (n = 46) (rehabilitation medicine, geriatric medicine, physiotherapy, occupational therapy, speech and language therapy, clinical psychology, podiatry, nursing, social work) was used to elicit the determinants of a BPS approach. Participants for this part of the study were recruited based on a purposive and criterion sampling strategy [32]. Healthcare professionals were recruited when they claimed to have experience in working according to a BPS model. Clients were recruited when they were treated within a BPS model. The clients were not recruited from the same institutions as the professionals to avoid participants being hindered in expressing their experiences due to existing patient-professional relationships. Data were gathered using an interview guide with open-ended questions. An independent and trained moderator, not involved in the institutions or in the research group, conducted the focus group interviews and started with an ‘ice breaker’ question: ‘If BPS practice is considered to be important in contemporary healthcare, encompassing biological, psychological and social aspect of human life, how do you apply this in your daily practice (professionals) / how did you experience this during your therapy (clients)’? Probes were used to deepen the answers and were used to get more insight in the research topic. Data was analysed by constantly comparing the data from the different focus groups and similarities between the experiences from the professionals and the clients were searched for. This process led to a list of items that represents a BPS approach. For instance: both patients and healthcare professionals expressed that when trying to include biological, psychological and social aspect into the process of health delivery, ‘the client should be considered in the goal making process and if possible, he should be invited to the team meetings’. Consequently, co-creation of goals and inviting clients to the team meeting were both considered to be items that represent aspects of a BPS approach. All items identified in this qualitative study were checked whether they reflects the BPS construct. Firstly, we checked whether the items were present and described as important aspects in current literature on BPS practice. For instance the item to set goals in close collaboration with the client is extensively described in literature from Wade when describing the implementation of a BPS model into practice [15]. Secondly, whether these items were considered to be true determinants of a BPS approach was reviewed by an independent team of scholars with research expertise in BPS healthcare delivery from the healthcare department of the University College PXL (occupational therapists, physiotherapists and social workers). Subsequently, after their validation, the items were rephrased into statements and again checked by the healthcare team. To increase the credibility of the answers, the items were rephrased into statements with the reference to their last client. An example of a statement: *‘related to your last client*: *I discussed my clinical decision with my colleagues’*. The respondents were asked to assess the different statements by means of a 5 point Likert scale ranging from (1) I totally disagree to (5) I totally agree.

### Step 2: factor structure and item reduction

#### Study sample for the scale

This study was carried out in Flanders, which is the Dutch speaking part of Belgium. All statements generated in step 1 were combined in a digital survey. The digital survey was sent out to healthcare and welfare organisations that are united under an umbrella organisation of Flemish organisations. These organisations provided a broad sample, covering different settings such as nursing homes, hospitals, community care, etc., ensuring a regional spread. The contact person within the setting was asked to pass the survey to a randomly selected healthcare professional in the organisation. The study was approved by the ethics committee of Ghent University Hospital, and by completing the survey all participants gave their consent. Note that the original scale was developed in Dutch. For publication, this scale was translated in English with a forward and a backward translation to ensure a conceptual equivalence and not a purely linguistic equivalence.

#### Factorial structure

An exploratory factor analysis was conducted to determine the factor structure of the scale and to see if there were subscales in the scale. A second reason for the factor analysis was to reduce the number of items. Maximum likelihood was used as the extraction method, and in order to maximise factor simplicity oblique rotation (promin) was used as a rotation method [33]. To check whether the data were appropriate to conduct the exploratory factor analysis, the Kaiser-Meyer-Olkin measure of sampling adequacy was performed, defined beforehand to be greater than 0.70 [34]. Additionally, Bartlett’s test of sphericity was performed, defined beforehand to be significant at the level of p<0.01 to ensure that the correlations that appeared in the dataset were appropriate for factor analysis [34]. In order to determine whether the sample size was adequate to yield distinct and reliable factors, the communalities after extraction were calculated, and should be above 0.60 [35,36]. Items not loading on a factor (<0.50) were deleted from the final scale. The factor analysis was performed multiple times with a restricted number of factors, and compared to each other to define the ideal-fit model.

#### Internal consistency

The internal consistency was expressed with a Cronbach’s alpha coefficient. The homogeneity is considered to be good if Cronbach’s alpha ranges between 0.70 and 0.95 [37]. Cronbach’s alpha was calculated for all the subscales. Furthermore, for each subscale an item-total correlation was calculated. Items not contributing to the internal consistency (item-total correlations under 0.4) were excluded from the final subscale.

### Step 3: reliability and clinical applicability

#### Test-retest reliability

The BPS scale (after data-reduction) was administered two times (T1 and T2) in a new randomly selected sample from one rehabilitation centre including different healthcare professionals. An interval of 1 week was used to calculate the test-retest reliability. An intra-class-correlation coefficient (ICC) according to the 2-way mixed method was used. The test-retest reliability was considered good if the ICC ≥ 0.70 [37].

#### Feasibility

The feasibility of the BPS scale was assessed by the time needed to complete the questionnaire. The digital platform did not allow answers to be left blank.

#### Scoring and interpretability

The scores of all participants for each statement were recalculated into mean scores to get one single score for each statement [38,39]. A total score was calculated by summarising the mean scores of all the statements for each subscale (which were derived from the factorial analysis), divided by the number of items.

**Fig 1:**
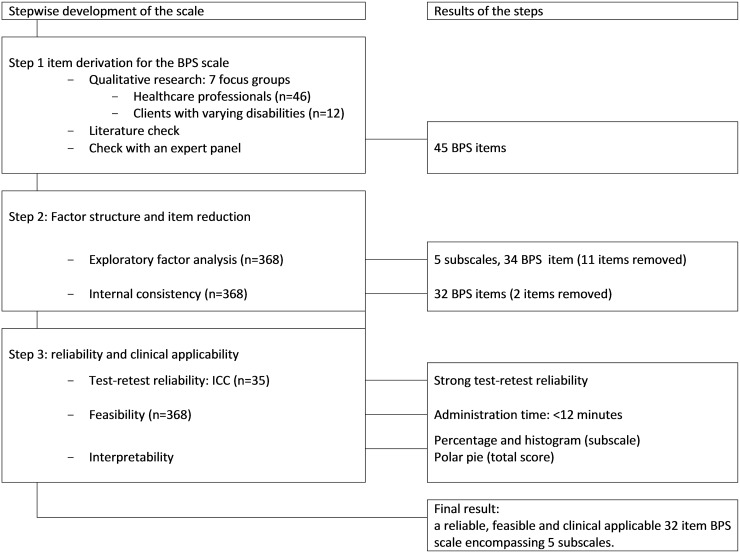
Flow chart of the development process of the BPS scale.

The exploratory factor analysis was conducted using Factor [40]. All other statistics were administered with SPSS 22 [41]. The entire process of the scale development process is presented in Fig 1.

## Results

### Step 1: item derivation

The qualitative study revealed 45 different items. All items discovered by means of the qualitative study could be maintained because (1) all items could be detected in the literature as important aspects of a BPS approach and (2) all aspects were considered relevant based on the expert opinions.

### Step 2: factor structure and item reduction

#### Study sample for the scale

Three hundred and sixty-eight healthcare professionals were included in the first sample, 17.6% male and 82.4% female, with a mean age of 39.1 years. The sample was heterogeneous regarding professional discipline and the region of Flanders. All specific characteristics are presented in Table 1. The second sample (n = 35) for the test-retest reliability comprises 7 nurses, 8 occupational therapists, 6 physical therapists, 4 speech and language therapists, 5 medical doctors, 1 psychologist and 4 social workers.

**Table 1:** Characteristics of the participants (n = 368).

Age: mean (SD, range)	39.1 (10.8)
Gender, n M/W	56/262
Years of experience in healthcare delivery n (%) range 1–42	
No experience (students)	50 (13.6)
Less than 5 years	51 (13.9)
Between 5 and 10 years	78 (21.2)
Between 10 and 20 years	93 (25.3)
More than 20 years	96 (26.1)
Disciplines (n %)	
Medical Doctor	27 (7.3)
Nurse (including midwifery)	66 (17.9)
Physiotherapist (including podiatry)	51 (13.9)
Speech and language therapist (including audiology)	64 (17.4)
Occupational therapist	125 (34.0)
Other (psychologist, cultural worker, gerontologist, orthoptist, social worker)	35 (9.5)
Setting (n %)	
Primary care	23 (6.3)
Inpatient rehabilitation setting adults	57 (15.5)
Outpatient rehabilitation center adults	60 (16.3)
Inpatient rehabilitation center children	28 (7,6)
Outpatient rehabilitation center children	29 (7.9)
Elderly care	82 (22.3)
Psychiatric care	39 (10.6)
Educational programme	50 (13.5)

#### Factorial structure

The Kaiser-Meyer-Olkin measure of adequacy was 0.82, and Bartlett’s test of sphericity was statistically significant (*x*^*2*^ = 856.2, *df* = 115, *p* < 0.01), meaning that the data were appropriate to conduct an exploratory factor analysis. Because communalities after extraction ranged from 0.61 to 0.82, our sample size of 368 could be considered adequate. The factor analysis was performed multiple times with a restricted number of factors (2, 3, 4, 5, 6 and 7 factors), and these structures were compared to each other. The factor analysis with a five factor solution which consequently led to a scale with five subscales that showed the ideal model fit. Each factor was given a name and labelled in mutual agreement with the different authors based on the content of the items. The labels of these factors became the labels of the different subscales. The first factor, *‘networking’* had 8 items and accounted for 16.45% of the total variance. The second factor, *‘using the expertise of the client’* had 5 items and accounted for 12.56% of the total variance. The third factor, *‘assessment and reporting’*, had 6 items and accounted for 11.84% of the total variance. The fourth factor, *‘professional knowledge and skills’*, had 11 items and accounted for 17.12% of the total variance. Finally, the fifth factor, *‘using the environment’*, had 7 items and accounted for 10.63% of the total variance. With this factor solution 68.6% of the total variance could be explained.

Nine items did not load on one of these five factors and two items loaded under 0.50. These 11 items were excluded from the final scale (see Table 2).

**Table 2:** Exploratory factor analysis: rotated loading matrix* (n = 368).

		Factor 1	Factor 2	Factor 3	Factor 4	Factor 5
		16.45%	12.56%	11.84%	17.12%	10.63%
1	I discussed the clinical decisions with my colleagues	**0.57**	-	-	-	-
2	I discussed the clinical decision with relevant stakeholders outside my organisation	**0.72**	-	-	-	-
3	Non-healthcare related professionals also had an important role in goal-setting for the client.	**0.56**	-	-	0.44	-
4	Healthcare professionals help each other in complex care needs.	**0.78**	0.45	0.52	-	-
5	The inter-professional cooperation in my team is good.	**0.82**	-		-	-
6	My superior is supportive when difficult decisions need to be taken.	**0.72**	-	-	-	-
7	I used the findings from my colleagues from other disciplines when listing this client’s problems.	**0.78**	0.54	-	-	-
8	The client was invited to the team meetings.	0.42	**0.56**	-	-	-
9	I used the lived experience of the client in clinical decision making.	-	**0.68**	0.33	-	-
10	I have informed my client about the clinical choices that were made.	-	**0.84**	-	-	-
11	My management offers me tools to enable a client-centred practice.	-	**0.62**	0.43		
12	The management in my unit is focused on formulating goals together with the client (shared goal-setting).	-	**0.63**	0.51	-	0.41
13	In our organisation the client is always the central point around which the therapy-plan evolves.	-	**0.52**	-	-	-
14	I have co-created the therapy goals with my client and/or his proxies.	-	**0.75**	0.42	-	0.37
15	I used assessment tools to monitor the client’s wishes.	-	0.58	**0.68**	-	-
16	I used assessment tools to monitor all levels of human functioning.	-	-	**0.54**	0.32	-
17	I have access to assessment tools to assess what the client finds important.	-	-	**0.69**	-	-
18	In my organisation we use a format of reporting that covers all aspects of human functioning.	-	-	**0.70**	-	-
19	I used my professional knowledge in my clinical decision making.	-	-	-	**0.72**	-
20	I used guidelines in clinical decision making.	-	-	-	**0.68**	-
21	*If my client doesn’t take responsibility for his goals*, *I make the goals for him*.**	0.36	-	0.45	0.56	-
22	*I feel insecure in my clinical reasoning because I’m not aware of the theoretical background*.**	-	0.35	-	0.58	0.37
23	I used my own professional experience in clinical decision making.	-	-	-	**0.72**	-
24	I have knowledge of different tools to assess what is important to the client.	-	-	0.54	**0.82**	-
25	I have the skills to approach my clients from a holistic point of view.	-	0.42	0.65	**0.73**	-
26	I have the skills to involve the family into the therapy process.	-	0.36	0.49	**0.67**	0.50
27	I have the skills to defend my clients’ choices in a team meeting	0.48	-	0.52	**0.64**	-
28	When formulating goals for the client, I considered the meaning of his family.	-	-	-	-	**0.74**
29	We invited the client (and his family) to discuss the therapy plan.	-	-	-	-	**0.65**
30	I worked in close collaboration with the client’s proxies.	-	-	-	-	**0.76**
31	I used the family’s contribution in clinical decision making.	-	-	-	-	**0.72**
32	I have treated my clients at home.	-	-	-	-	**0.51**
33	I have used information about the familiar home-environment to make clinical decisions.	-	-	-	-	**0.50**
34	My management endorses me to visit and treat the client in his familiar home-environment.	-	-	-	-	**0.57**
35	*I have the skills to reason on my own without considering the other disciplines*.***	0.32	-	0.42	0.47	0.38
36	*I consider my task as very complex*.***	0.35	0.32	-	0.45	0.39
37	*I’m aware of the pros and cons of working with a biomedical model*.***	-	-	-	-	-
38	*I’m aware of the pros and cons of working with a bio-psycho-social model*.***	-	-	-	-	-
39	*I rely more on my professional knowledge*, *than on my experience*.***	-	-	-	-	-
40	*I made decisions on my own without considering the other healthcare professionals*.***	-	-	-	-	-
41	*I find my way of clinical reasoning to be more effective than the reasoning process of my colleagues*.***	-	-	-	-	-
42	*Communication with the client is part of the ‘on -the job-training’*.***	-	-	-	-	-
43	*We are trained on-the-job to enhance our clinical reasoning skills*.***	-	-	-	-	-
44	*The medical staff is only concerned with biomedical aspects of human functioning*.***	-	-	-	-	-
45	*I’m more skilled in working with people than in working with theoretical models*.***	-	-	-	-	-

Labels of the factors: factor 1: networking; factor 2: using the expertise of the client; factor 3: assessment and reporting, factor 4: professional knowledge and skills, factor 5: using the environment.

* Scores beneath absolute 0.30 omitted to increase the readability.

** Items left out of the final scale (n = 2) based on a low item total correlation (see internal consistency).

*** Items not loading (n = 9) or loading under 0.50 (n = 2) on one of the five factors are left out of the final scale.

Items in italics were eventually removed from the final BPS scale (n = 13).

#### Internal consistency

The analysis showed that the homogeneity of the subscales 1, 2, 3 and 5 showed a fair to strong item-total correlation ranging from 0.57 to 0.86 and an acceptable to high Cronbach’s α ranging from 0.75 to 0.82 (Table 3). The fourth subscale of *‘professional knowledge and expertise’* could be improved by removing two items: *‘If my client doesn’t take responsibility for his goals*, *I make the goals for him* (item-total correlation 0.36) and *‘I feel insecure in my clinical decision-making because I’m not aware of the theoretical background* (item-total correlation 0.35) (see Table 2, items 21, 22). Removing these items resulted in an increase of Cronbach’s α from 0.56 to 0.78 (see Table 3).

**Table 3:** Internal consistency: Cronbach’s α for each subscale before and after item-reduction (n = 368), test-retest reliability comparing T1 with T2: ICC on scale level (n = 35).

	Cronbach’s α Before (and after) item reduction	ICC**	Confidence interval ICC
Subscales based on the exploratory factor analysis			
Networking	0.81	0.82	0.76–0.91
Using the expertise of the client	0.75	0.88	0.74–0.89
Assessment and reporting	0.82	0.88	0.81–0.95
Professional knowledge and skills*	0.56 (0.78)	0.92	0.82–0.97
Using the environment	0.76	0.93	0.81–0.96

* Two items were removed from this subscale.

** ICC: intra-class correlation coefficient.

In total, 13 items were removed from the final scale: 11 items by means of the factor analysis, and 2 items by means of the low item total correlation. The final scale exists out of 32 items divided into 5 subscales.

### Step 3: reliability and clinical applicability

#### Test-retest reliability

The test-retest reliability was performed in a new randomly selected sample (n = 35), comprising 7 nurses, 8 occupational therapists, 6 physical therapists, 4 speech and language therapists, 5 medical doctors, 1 psychologist and 4 social workers. When calculating the test-retest reliability comparing T1 with T2, the ICC ranged from 0.82 to 0.93 indicating a good test-retest reliability for every subscale (see Table 3).

#### Feasibility

The average administration time of the entire survey was 12 minutes (SD 3 minutes).

#### Scoring and interpretability

**Fig 2:**
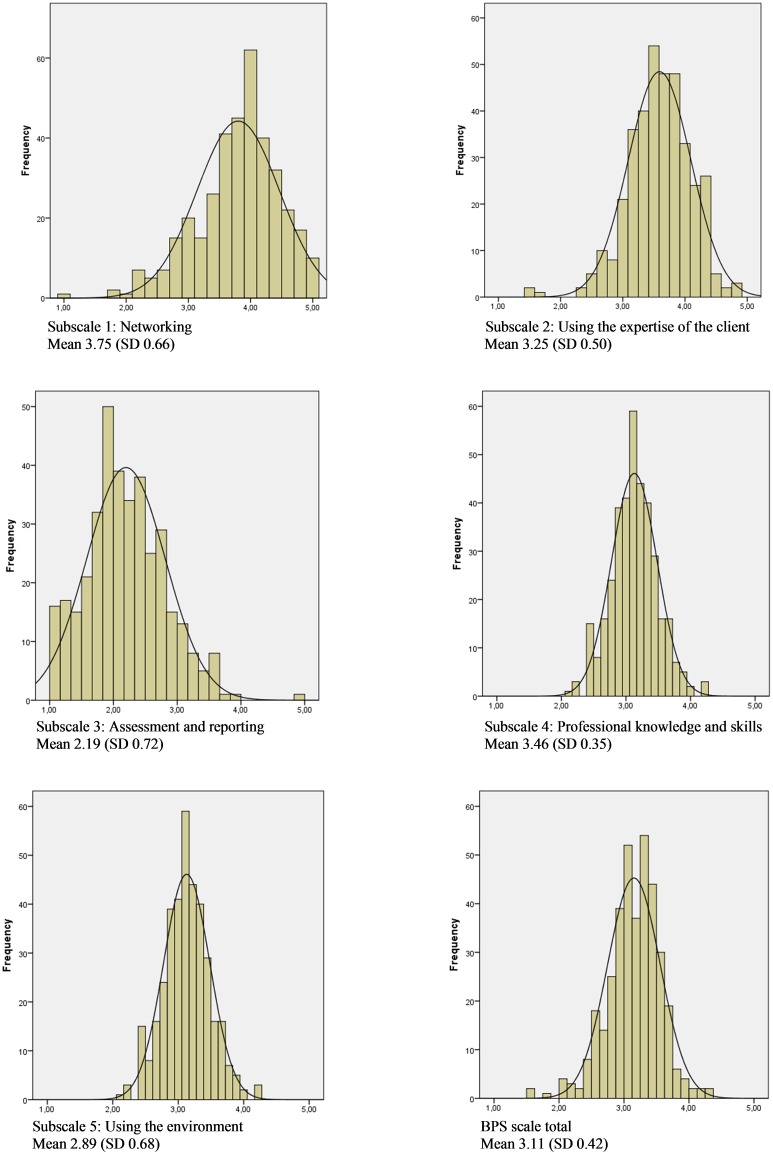
Histograms of the different subscales, displaying the normal curve (n = 368).

**Fig 3:**
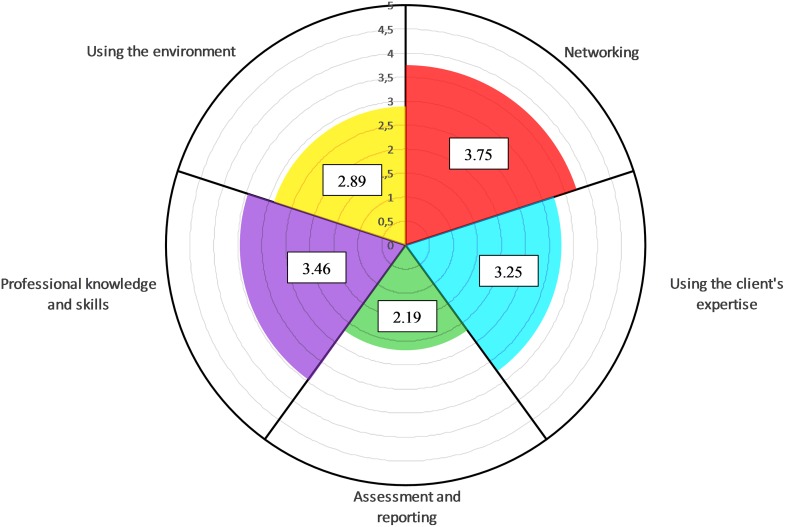
Graphical representation of the results of the BPS scale in a polar pie divided per subscale (n = 368), min. score: 1, max. score: 5.

The highest score is for the subscale ‘*networking’* (3.75, SD 0.66), followed by the subscales *‘professional knowledge and skills’* (3.46, SD 0.35), *‘using the expertise of the client* (3.25, SD 0.50) and *‘using the environment’* (2.89, SD 0.68). The lowest score is for the subscale *‘assessment and reporting’* (2.19, SD 0.72). The distribution of the total score and the scores on the different subscales seemed reasonable based on the skewness figures and the small difference between the mean scores and the median score (see Table 4 and Fig 2). To interpret the results, the scores of the scale, the subscales and the individual items were multiplied by 20 to get an indication in percentage. Additionally, to increase the interpretability, the scores of the different subscales were presented in a polar pie (see Fig 3).

**Table 4:** Scores on the BPS scale, the underlying subscales and on item level (n = 368).

	Mean (SD*)	Median (IQR**)	Min-Max	%floor	%ceiling	Skewness	Percent
Subscale 1: Networking	3.75 (0.66)	3.80 (0.80)	1.00–5.00	0.3	2.7	-0.69	75.0
I discussed the clinical decisions with my colleagues.	3.89 (0.04)	4.00 (1.00)	1.00–5.00	5.0	25.4	-1.24	77.8
I discussed the clinical decision with relevant stakeholders outside my organisation.	3.45 (1.10)	4.00 (1.00)	1.00–5.00	4.7	9.7	-0.61	69.0
Non-healthcare related professionals also had an important role in goal-setting for the client.	3.40 (1.22)	4.00 (1.00)	1.00–5.00	6.3	13.5	-0.44	68.0
Healthcare professionals help each other with patients with in complex care needs.	3.57 (0.61)	4.00 (1.00)	1.00–5.00	5.8	14.2	-0.74	71.4
The inter-professional cooperation in my team is good.	4.06 (0.75)	4.00 (1.00)	1.00–5.00	0.7	22.0	-1.11	81.2
My superior is supportive when difficult decisions need to be taken.	3.82 (0.42)	4.00 (1.00)	1.00–5.00	14.3	9.8	-0.65	67.4
I used the findings from my colleagues from other disciplines when listing to the client’s problems.	4.07 (0.49)	4.00 (1.00)	1.00–5.00	9.2	11.5	-1.07	81.4
Subscale 2: Using the expertise of the client	3.25 (0.50)	3.66 (0.67)	1.50–4.83	0.0	0.0	-0.53	65,0
I used the lived experience of the client in clinical decision making.	2.71 (0.45)	3.00 (1.00)	1.00–5.00	5.8	1.3	-0.54	54.2
I have informed my client about the clinical choices that were made.	3.30 (0.72)	4.00 (1.00)	1.00–5.00	0.2	28.1	-0.99	66.0
The client was invited to the team meetings.	3.22 (0.06)	3.00 (2.00)	1.00–5.00	1.6	20.7	-0.32	64.4
My management offers me tools to enable a client-centred practice.	3.12 (0.91)	4.00 (1.00)	1.00–5.00	1.3	25.4	-1.19	62.4
The management in my unit is focused on formulating goals together with the client (shared goal-setting)	3.71 (0.94)	4.00 (1.00)	1.00–5.00	1.4	11.5	-0.77	74.2
In our organisation the client is always the central point around which the therapy-plan evolves.	3.01 (1.09)	3.00 (1.09)	1.00–5.00	7.6	5.4	0.02	60.0
I have co-created the therapy goals with my client and/or his proxies.	3.70 (0.98)	4.00 (1.00)	1.00–5.00	1.4	14.1	0.13	74.0
Subscale 3: Assessment and reporting	2.19 (0.72)	2.00 (1.00)	1.00–5.00	0.3	3.0	0.25	43.9
I used assessment tools to monitor the client’s wishes.	2.05 (0.32)	2.00 (1.00)	1.00–5.00	12.2	10.5	0.35	41.0
I used assessment tools to monitor all levels of human functioning	2.12 (0.99)	2.00 (2.00)	1.00–5.00	20.0	0.9	0.69	42.4
I have access to assessment tools to assess what the client finds important.	2.76 (0.83)	2.00 (1.00)	1.00–5.00	3.8	0.9	0.01	55.2
In my organisation we use a format of reporting that covers all aspects of human functioning.	2.02 (0.45)	2.00 (1.00)	1.00–5.00	6.3	0.00	0.05	40.4
Subscale 4: Professional knowledge and skills	3.46 (0.35)	3.89 (0.44)	2.11–4.22	0.0	0.0	-0.23	69.4
I used my professional knowledge in clinical decision making.	3.97 (0.49)	4.00 (1.00)	1.00–5.00	1.1	23.8	-0.96	79.4
I used guidelines in my clinical decision making.	3.20 (1.20)	4.00 (2.00)	1.00–5.00	6.1	9.7	-0.20	64.0
I used my own professional experience in clinical decision making.	3.56 (0.88)	4.00 (1.00)	1.00–5.00	9.9	1.4	-0.14	71.2
I have knowledge of different tools to assess what is important to the client.	3.45 (1.10)	4.00 (2.00)	1.00–5.00	4.7	9.7	-0.61	69.0
I have the skills to approach my clients from a holistic point of view.	3.00 (1.09)	3.00 (2.00)	1.00–5.00	7.6	5.4	-0.05	60.0
I have the skills to involve the family in the therapy process.	3.96 (0.81)	4.00 (0.00)	1.00–5.00	0.7	15.9	-0.88	79.2
I have the skills to defend my clients’ choices in a team meeting.	3.14 (1.18)	4.00 (2.00)	1.00–5.00	7.0	8.3	-0.21	62.8
Subscale 5: Using the environment	2.89 (0.68)	3.01 (0.83)	1.00–4.83	0.5	0.0	-0.22	57.8
We invited the client (and his family) to discuss the therapy plan.	3.23 (1.27)	4.00 (2.00)	1.00–5.00	9.2	11.5	-0.32	64.6
I worked in close collaboration with the client’s proxies	2.90 (0.94)	3.00 (1.00)	1.00–5.00	6.1	3.2	-0.12	58.0
I used the family’s contribution in clinical decision making.	3.05 (0.88)	4.00 (1.00)	1.00–5.00	0.7	11.7	-0.63	61.0
I have used information about the familiar home-environment to make clinical decisions.	2.56 (1.35)	2.00 (3.00)	1.00–5.00	19.5	7.6	0.39	51.2
I have met the client in his familiar home-environment.	3.15 (1.21)	4.00 (1.00)	1.00–5.00	7.4	11.5	-0.48	63.0
My management endorses me to visit and treat the client in his familiar home-environment.	2.47 (1.30)	2.00 (2.00)	1.00–5.00	21.5	5.8	0.41	49.4

* Standard Deviation

** Inter Quartile Range

## Discussion and Conclusion

Since the shift from a purely biomedical perspective to a BPS model has become apparent in healthcare literature, healthcare professionals are expected to work accordingly [42–44]. This means that healthcare professionals are asked to take into account all aspects of human functioning in their clinical work. Unfortunately, this is not fully the case in contemporary practice. Healthcare professionals have to deal with new theories and new approaches as a result of this shift. This shift means that the health care professionals need other knowledge and skills [12,15]. It shows at the same time the gap between the rhetoric and the reality of working within a BPS model [16]. Therefore, our group set out to develop a BPS scale to rate the level of BPS practice in healthcare. It was specifically the goal of this study to focus on the process and the skills of the healthcare professional.

A strong point in this study is the fact that the instrument was developed based on the experiences of a wide variety of both expert-professionals and clients using a qualitative research method. Additionally, by checking these items with the body of knowledge from recent literature, the credibility of the scale has been increased. By combining these two methods we were able to operationalise the process of working according to a BPS model in such way that health care professionals could rate their own BPS competencies in a valid and reliable way.

The factor analysis revealed that the scale could be divided in five subscales. Eleven from the 45 items showed no statistical coherence with any of the five subscales, and were left out of the final scale. At first it seemed remarkable that these 11 items did not fit within the scale because of the strong assumption, based on the item generation method, that all of these items were relevant for rating the level of BPS practice. However, three reasons for this are hypothesised: (1) some questions were phrased too much from a theoretical point of view and started from a presumption that the respondent has a priori theoretical knowledge, for instance: ‘*I’m aware of the pros and cons of working with a bio-psycho-social model’*; (2) some questions contained a value judgement that perhaps made it difficult for the respondent to give a sincere answer, for instance: *‘the medical staff is only considered with biomedical aspects of human functioning’;* and (3) these items could be stand-alone items, meaning that there were no other items in the list measuring the same ‘construct’. Additionally, two items were removed because they were not very consistent with the total score of the subscale (item-total correlation below 0.30).

The feasibility of the scale is reasonable to good. Before data reduction it took 12 minutes to administer the scale. It is expected that with the reduction of 13 items leading to the final scale of 32 items the administration time will be slightly reduced (approximately 9 minutes).

The test-retest reliability was strong to excellent on the scale level (ICC range from 0.82 to 0.93). The participants scored higher on average on T2 in comparison with T1 on the item *‘in my organisation we use a format of reporting that covers all aspects of human functioning’*. There is no specific explanation for why this average score is higher. However, it could be that by answering the questions at T1, attention was drawn to the topic, and consequently, the participant was more focused on the topic; therefore, he/she was more compelled to use the existing formats for reporting, even though he/she did not do that before. It is commonly known that studies do interfere with daily practice.

The BPS scale allows the individual healthcare worker and the healthcare organisation to visualise the BPS competencies of the entire organisation or for the individual. This feature enhances its interpretability. By means of a polar pie (see Fig 1, showing the results for the entire scale and the five subscales) and histograms (see Fig 2, for each subscale), it is easy to detect the strong and weak aspects of a BPS practice. Having an easy-to-interpret graphical representation of these results is an advantage and creates opportunities for the organisations to launch improvement projects, which are of paramount importance in quality improvement and assurance in healthcare delivery [45].

The average score of the total BPS scale is 3.11 on a scale from 1 to 5—at first sight a rather good score. However, the ‘ideal BPS practice’ has not yet been reached. Looking more in detail at the results, a number of points for improvement for BPS practice have evolved.

As an example, in the results of this sample, the subscale *‘assessment and reporting’* has the lowest score (2.19, SD 0.72), indicating that the healthcare professionals fail in assessing the client on all levels of human functioning, on reporting in a coherent way, and consequently, also on integrating it into the whole cycle of the process. These aspects, however, are considered to be of utmost importance in BPS practice for different reasons; (1) an accurate assessment is necessary to identify all relevant factors to identify goals and sub-goals [12] and (2) it helps in liaising with all other team-members, with other teams and with other organisations [46].

As another example, the subscale ‘*using the environment’* (2.89, SD 0.68) also shows that there are opportunities for improvement in the BPS practice with regard to involving the environment. This is particularly important because research has shown that involving the environment has a positive effect on the process of healthcare delivery. The subscale ‘*using the clients’ expertise’* has a score of 3.25 (SD 0.50) and refers to extent of the client-centred attitude of the healthcare professional, which also clearly shows opportunities for improvement. For instance, the item ‘*using the clients’ lived experience in clinical decision making’* has a score of merely 2.71 (SD 0.50), which is rather weak according to the premise that a client-centred approach entails the use of this knowledge to improve the quality of care [10]. The subscale *‘professional knowledge and skills’* has a score of 3.46 (SD 0.35) and shows a smaller range (2.11–4.22) in comparison with the other subscales. The absence of extreme values of 0 and 5 indicate that there are no individuals claiming to have extreme (in)-sufficient skills and knowledge for an ideal BPS practice. This aspect itself is a sufficient argument to organise courses on underlying theories and skills training. Finally, the subscale *‘networking’* obtained a score of 3.75 (SD 0.66), which is the highest score in relation to the other subscales.

The median of nearly 4 shows that the overall interdisciplinary work is rated as very good. A strong interdisciplinary collaboration is favourable in a BPS practice. Research has shown BPS to be effective in different pathology groups such as, for instance, multiple sclerosis [47], or acquired brain injury [48]. However, there are also significant opportunities for improvement here when looking at the scores of the different items.

### Limitations of the study and future research

Firstly, with regard to the sample size of 368 participants, this could be considered as small when following the ‘subject to item ratio of 10:1’ as a rule-of-thumb in performing factor analysis [49]. That means that the minimum sample size for an analysis should be 10 cases for each variable and in this study, the need for a sample size of at least 450 (45 items before item reduction). However, the guidelines from MacMallum et al. [36] and Henson et al. [37]—in which a smaller sample is accepted when the communalities after extraction are above 0.60—was followed, since the communalities after extraction ranged between 0.61 and 0.82. Therefore, the sample-size was considered adequate, although a follow-up study in a broader sample might be advisable. It is, however, necessary to confirm the factor structure by running a confirmatory factor analysis in another sample.

Secondly, the test-retest reliability was performed in a small sample. Although the results were good with an ICC ranging from 0.82 to 0.93 and look promising, a more profound study in a larger sample is necessary.

Third, whether the instrument will be able to detect changes over time in quality improvement projects is not yet known but is absolutely necessary since the goal of this instrument is to improve healthcare delivery. Consequently, further research on the responsiveness of the BPS scale is ongoing.

Fourth, in social science, it is well-known that respondents tend to answer socially desirable responses and to answer from a general instead of personal perspective. This scale is specifically constructed to prevent these phenomena. In the question-construction about knowledge, skills and support for a BPS practice, all questions were related to the ‘last patient’ they have treated. Moreover, the statements were formulated in the ‘I’-form.

Fifth, there is a need to conduct a study to test the construct validity of the scale by correlating the subscales with instruments measuring the different constructs within the scale.

Finally, the information about the disciplines (e.g. nurses, occupational therapists, etc.) and the settings (e.g. primary healthcare, rehabilitation center, etc.) was only meant to be exploratory and needs further research to explore in depth the possible differences in BPS practice between different healthcare professionals. The BPS scale is intended to be healthcare professional independent, so there was no a priori expectation about possible differences in BPS practice between the different groups. Moreover, the different subgroups of healthcare professionals in this sample are too small to make conclusions. Additionally, this scale has been developed in the Dutch speaking part of Belgium and only Dutch speaking healthcare professionals were included. Further research is necessary to establish further applicability in healthcare professionals from other countries and other healthcare cultures.

This study, describing the development and validation of a BPS scale, adds, in a way, to the debate among scholars about the need and the search for a new definition of health. It is commonly known that in contemporary discourse on the concept of health, it is no longer defined as *‘a state of complete physical*, *mental and social wellbeing and not merely the absence of disease’* [50]. Instead the discourse now is shifting to defining health as *‘the ability to adapt to the environment’* [51]. A seemingly new way of thinking, if were not for the fact that this definition was described for the first time in 1943 by Canguilhem [52]. Nowadays, the discourse has been picked up again and researchers and scholars have elaborated further on this conceptual idea. Huber and colleagues, for instance, propose to evolve towards *‘the ability to adapt and self-manage’* [53]. This definition is not a generally accepted one and is somehow controversial. Kruseman [54], for instance, argues against this latter definition by combining the best elements of the old and the new definition. According to him, the truth is somewhere in the middle of the spectrum between the ability to adapt and experiencing well-being. Therefore, he concludes that health is *‘the ability to adapt and self-manage resulting in physical*, *mental and social well-being’*. Looking at health from this perspective it is clear that health professionals are in need of a set of competencies to tackle this paradigm shift, since a different way of approaching the client is advisable. These competencies are in fact the BPS competencies: (1) being able to network, (2) using the expertise of the client, (3) being capable for assessment and reporting, (4) having specific professional knowledge and skills and (5) using the environment.

In this changing healthcare world, healthcare professionals and organisations should be aware of where they stand in the quality of care. The BPS scale, focusing on the client, his expertise, his experiences, his environment and his goals, combined with the professional knowledge from the healthcare professionals and the support from the management, could fill the gap in the challenge to improve healthcare delivery by shifting from the biomedical towards a BPS paradigm. This scale however only provides insight and awareness in their skills and competencies. The use of a framework such as the ICF might be necessary to give health care professionals also a framework and a language for daily practice, enhancing transparent communication.

## Conclusion

In order to enhance practitioners’ effectiveness in reasoning and acting according to a BPS model in daily practice, a reliable, feasible, valid and timely instrument to rate the level of BPS practice—applicable for all healthcare professionals and for the entire healthcare system—was developed and validated. The instrument is in accordance with the contemporary health paradigm and offers opportunities to improve the quality of the healthcare delivery based on five subscales: (1) the competencies and the support in networking, (2) the level of involving the client in the process, (3) the level of using professional knowledge and skills, (4) the level of assessment and the coherent way of reporting and finally, (5) the competence to use the environment in clinical decision making. With this scale, the level of BPS practice can be interpreted by means of alphanumeric scores, a polar pie and histograms. This scale was developed to collect data in a feasible way, to detect issues in BPS practice and to define improvement plans. To this end, having knowledge of these issues is an advantage, which significantly increases the chance that improvement measures will succeed.

## Appendix: The Bio-Psycho-Social Scale

By answering the questions, please keep your last client in mind.

Response options for all items is each subscale: 1: I totally disagree to 5: I totally agree

### Subscale 1: Networking

1. I discussed the clinical decisions with my colleagues.
2. I discussed the clinical decision with relevant stakeholders outside my organisation.
3. Non-healthcare related professionals also had an important role in goal-setting for the client.
4. Healthcare professionals help each other when patients have complex care needs.
5. The inter-professional cooperation in my team is good.
6. My superior is supportive when difficult decisions need to be taken.
7. I used the findings from my colleagues from other disciplines when listing to this client’s problems.

### Subscale 2: Using the expertise of the client

1. I used the lived experience of the client in clinical decision making.
2. I have informed my client about the clinical choices that were made.
3. The client was invited to the team meetings.
4. My management offers me tools to enable a client-centred practice.
5. The management in my unit is focused on formulating goals together with the client (shared goal-setting).
6. In our organisation the client is always the central point around which the therapy-plan evolves.
7. I have co-created the therapy goals with my client and/or his proxies.

### Subscale 3: Assessment and reporting

1. I used assessment tools to monitor the client’s wishes.
2. I used assessment tools to monitor all levels of human functioning.
3. I have access to assessment tools to assess what the client finds important.
4. In my organisation we use a format of reporting that covers all aspects of human functioning.

### Subscale 4: Professional knowledge and skills

1. I used my professional knowledge in clinical decision making.
2. I used guidelines in my clinical decision making.
3. I used my own professional experience in clinical decision making.
4. I have knowledge of different tools to assess what is important to the client.
5. I have the skills to approach my clients from a holistic point of view.
6. I have the skills to involve the family into the therapy process.
7. I have the skills to defend my clients’ choices in a team meeting.

### Subscale 5: Using the environment

1. We invited the client (and his family) to discuss the therapy plan.
2. I worked in close collaboration with the client’s proxies.
3. I used the family’s contribution in clinical decision making.
4. I have used information about the familiar home-environment to make clinical decisions.
5. I have met the client in his familiar home-environment.
6. My management endorses me to visit and treat the client in his familiar home-environment.
